# From lonely to addicted: exploring sex differences in the effect of online social support among university students in Hong Kong

**DOI:** 10.3389/fpsyg.2025.1654908

**Published:** 2025-11-06

**Authors:** Mei-Kei Leung, Kean Poon, Nichol M. L. Wong, Hon-Cheung Lam, Way Kwok-Wai Lau

**Affiliations:** 1 Department of Counselling and Psychology, Hong Kong Shue Yan University, Hong Kong, Hong Kong SAR, China; 2 School of Education, The University of New South Wales, Sydney, NSW, Australia; 3 Department of Psychology, The Education University of Hong Kong, Hong Kong, Hong Kong SAR, China; 4 Department of Diagnostic Sciences, Hong Kong Metropolitan University, Hong Kong, Hong Kong SAR, China

**Keywords:** loneliness, internet addiction, perceived online social support, sex differences, mediation analysis, psychosocial

## Abstract

**Introduction:**

Internet addiction, a growing issue in young adults post-pandemic, especially among Asian males, is linked to various negative outcomes. Loneliness significantly influences internet addiction, but the underlying psychosocial mechanism remains unclear. This study examined the mediating role of online social support between loneliness and internet addiction with a focus on sex differences.

**Methods:**

A cross-sectional online survey of 213 university students (105 males and 108 females), recruited through convenience sampling, examined loneliness, internet addiction, and perceived online social support.

**Results and discussion:**

No significant moderation effect of sex on the direct or indirect path between loneliness and internet addition. Perceived online social support partially mediated the positive association between loneliness and internet addiction only in males [standardized *β* = 0.07, bootstrapped SE = 0.04, bootstrapped 95% CI (0.01, 0.17)], but not in females [*β* = 0.03, bootstrapped SE = 0.03, bootstrapped 95% CI (−0.02, 0.11)]. On the other hand, perceived online social support was significantly and positively correlated with internet addiction in both sexes [standardized *β* = 0.27, bootstrapped SE = 0.10, 95% CI (0.07, 0.47)]. Our findings reveal a potential concern of online social support, which could be one of the underlying psychosocial factors contributing to a deeper reliance on the internet and further social withdrawal from the real world in males. This deserves more in-depth investigations regarding the influence of different types of online social support received or perceived through different online platforms and environments. The findings of this study have implications for how online social support is structured for individuals struggling with internet addiction, especially male individuals, and underscore the need for gender-sensitive design in future online support programs.

## 1 Introduction

The use of the internet has dramatically increased over the past two decades, with a marked 31.7% rise in the amount of internet users worldwide since the onset of the COVID-19 pandemic, from 4.1 billion internet users in 2019 to 5.4 billion in 2023, according to the report of the International Telecommunication Union (ITU) of the United Nations (International Telecommunication Union, 2023). This surge, while offering numerous benefits, has also escalated the risks associated with excessive internet use, including the development of internet addiction or problematic internet use, which has reached a prevalence rate of 15.3% in southern China (Chi et al., 2020). According to some recent evidence, internet addiction continues to be a growing issue in the post-pandemic era in young adults around the world (Lozano-Blasco et al., 2022), especially for Asian males (Su et al., 2019), due to the increased exposure to risks of internet addiction, such as increased use of digital media (Fineberg et al., 2022).

Internet addiction is defined as the inability to control internet use that eventually leads to impaired psychological and social functioning, interpersonal relationships, academic and/or work performance (Li et al., 2016). Internet addiction is linked to a host of negative outcomes, including diminished academic and occupational performance, social isolation, and mental health issues such as anxiety, depression, and sleep disturbances (Cheng and Li, 2014; Kim et al., 2022). Among various risk factors, loneliness has consistently emerged as a significant predictor of internet addiction, underscored by a growing body of research (Moretta and Buodo, 2020). However, the underlying mechanisms that explain this relationship remain inadequately understood. Exploring the association between loneliness and internet addiction offers the potential to enhance both theoretical understanding and the development of practical interventions to mitigate the adverse effects of internet addiction.

## 2 Literature review

### 2.1 The relationship between loneliness and internet addiction

Loneliness, characterized as a negative, subjective experience stemming from unmet social needs and a lack of meaningful social connections (Peplau and Perlman, 1982), poses significant risks, particularly for young emerging adults. This demographic, being in a critical stage of social and emotional development and a transitional period between adolescence and full adulthood (Arnett, 2006), is especially susceptible to the detrimental effects of prolonged loneliness. Such effects include an increased risk of depression, anxiety, and lowered self-esteem, all of which can further exacerbate social withdrawal and isolation. While poor adjustment during this transitional period may pose a risk to a person’s future wellbeing (Nelson and Padilla-Walker, 2013), the COVID-19 pandemic has further intensified these challenges by disrupting traditional social structures and reducing in-person interactions. Consequently, many individuals have turned to the internet as a coping mechanism to alleviate loneliness (Shapira et al., 2000). A recent meta-analysis confirmed that there is a moderate positive association between loneliness and internet addiction (Wang and Zeng, 2024). Moreover, a previous longitudinal study revealed that loneliness exerts a greater and more extensive effect on internet addiction than the reverse (Zhang et al., 2018). Collectively, these findings clearly demonstrated the detrimental impact of loneliness in fostering problematic internet use.

### 2.2 The relationship between loneliness and online social support

Lonely individuals tend to spend more time online, seeking to maintain relationships and expand their social networks in the virtual world, in contrast to offline social networks in the real world. Specifically, studies indicated that the internet provides a platform for lonely individuals to curate their self-presentation (Walther, 2007) and navigate social interactions with reduced fear of rejection (LeFebvre, 2018).

Many studies suggested that lonely people could view the real-life social world as threatening based on a previous empirical review (Hawkley and Cacioppo, 2010), and lonely young adults can recall negative in-person social events more easily (Teneva and Lemay, 2020). The internet provides an additional communication channel to maintain relationships and a wider yet hidden network. Online communication allows individuals to carefully embellish their initial impressions (Walther, 2007) and craft their messages (Walther, 1996). Rejection cues in online communication are often obscure, making rejection more ambiguous (LeFebvre, 2018). Some studies indicated that lonely people alleviated socialization anxiety and psychological burden of rejection via online platforms and perceived such platforms as a supportive social atmosphere (Kehrwald, 2008; Kim and Timmerman, 2018). Further, online gaming is more prevalent among young males, another example. Online gaming emphasizes peer support on the internet and the interaction among players in the online community (Yu et al., 2021), which was seen as a form of social support that may at least partly explain why lonely people could be deeply immersed in internet use.

### 2.3 The dual impact of online social networks

While internet use can temporarily mitigate feelings of loneliness by fostering a sense of connection, it also carries the risk of leading to internet addiction. This complex relationship between loneliness and the potential outcomes of internet use highlights the necessity to unravel the intricacies of these dynamics, particularly in the context of a post-pandemic world.

If perceived online social support functions similarly to real-life support, lonely individuals who find solace in digital interactions may experience reduced internet addiction. However, if online support operates through a different mechanism, it might increase the likelihood of internet addiction. In fact, perceived online social support, defined as an individual’s belief that they have access to supportive relationships through digital platforms, differs from traditional, in-person social support in several ways. While online social support offers greater accessibility and anonymity, it may not fully replicate the quality of in-person relationships, potentially affecting its efficacy in preventing internet addiction. Therefore, more research is necessary to understand the role of perceived online social support in the loneliness-internet addiction relationship.

### 2.4 Sex differences in internet addiction

Sex differences are one of the most crucial demographic factors that affect the course and development of internet addiction (Su et al., 2020; Tang et al., 2017) and await further scrutiny. Although mounting evidence showed that internet addiction is more common in young men than women (Li et al., 2018), there are still some other evidence suggesting that young women, when comparing with young men, have higher prevalence of internet addiction (Al-Khani et al., 2021) or no sex differences on the prevalence rates of internet addiction (Holdoš, 2017), which altogether reflect how intricately sex may play a role in the development of internet addiction. On the other hand, a recent large-scale meta-analysis involving thirty-four countries still suggested that males are more likely to develop internet addiction than females in general, but these global sex differences on internet addiction are more severe in Asian population (Su et al., 2019), which could be resulted from Asian cultures that fail to deal with internet addiction effectively (Young, 2017). In fact, the prevalent rate of internet addiction in male adolescents in Hong Kong was consistently higher than that in female adolescents (Shek and Yu, 2016), suggesting the need to design an effective sex-specific approach for preventing and ameliorating internet addiction in teenagers and young adults in Hong Kong and beyond.

To achieve this, it is important to recognize that the psychosocial mechanisms underlying the development of the loneliness-internet addiction relationship may be different between sexes. Recent meta-analytic evidence provided strong support for the fact that the relationship between loneliness and internet addiction is generally moderated by sex (Wang and Zeng, 2024). Furthermore, males and females may become addicted to internet use through different pathways (Su et al., 2020). One of the earliest studies examining the role of online social support revealed that online social support was positively associated with internet addiction in both young males and females in Taiwan (Yeh et al., 2008). Wang and Wang (2013) added that this positive relationship between online social support and internet addiction was stronger for males than for females. In other words, they not only engage with online platforms differently but also derive different levels of emotional fulfillment from these interactions, which can differentially influence their susceptibility to internet addiction and the mechanism through which potential mediators like perceived online social support may manifest in internet addiction.

### 2.5 Research question and hypothesis

How sex may interfere with the inter-relationship between perceived online social support, loneliness, and internet addiction remains largely unexplored. The answer to this question will add an extra layer for the comprehensive understanding of how loneliness is linked to internet addiction through the influences of perceived online social support in young men and women.

The present study aimed to investigate the relationship between loneliness, perceived online social support, and internet addiction among young adults with a particular focus on sex differences. Grounded in the Compensatory Internet Use Theory that individuals experiencing loneliness may turn to the internet to seek social support, which can lead to excessive use or addiction (Kardefelt-Winther, 2014), this research examined how loneliness correlated with internet addiction and whether perceived online social support acted as a mediator in this relationship. Additionally, the study explored the influence of sex on these associations. Based on prior research, we hypothesized that perceived online social support mediated the relationship between loneliness and internet addiction in ways that differ from in-person interactions. Given that males and females may become addicted to internet use through different pathways and a higher prevalence rate of internet addiction in males in Hong Kong (Shek and Yu, 2016), we also hypothesized that the impact of perceived online social support on the connection between loneliness and internet addiction was stronger in male than female university students in Hong Kong.

## 3 Methods

### 3.1 Participants and procedures

This study adopted a cross-sectional design. University students aged 17 years and above were recruited using a convenience sampling method via a number of social media platforms such as Instagram, WhatsApp, and Facebook, as well as email circulation in the university. A minimum sample size of 158 participants was required based on a univariate linear regression model that predicted internet addiction with the following criteria: power = 0.95, effect size (*f*^2^) = 0.10, three total predictors in the model (loneliness, online social support, and age), and two tested predictors (loneliness and online social support). A total of 214 university students were recruited for this study.

All participants provided online informed consent before participating in the study. They filled in a set of questionnaires in Google Form measuring their levels of loneliness, perceived online social support, and internet addiction level from July 2022 to Aug 2023. The study procedures were approved by the Departmental Subcommittee and recorded in the Human Research Ethics Committee of Hong Kong Shue Yan University, Department of Counselling and Psychology (Ethics Reference Number: PSY371_188047_LHC_2022). All experiments were performed in accordance with relevant guidelines and regulations, including ensuring confidentiality and anonymity. The participation was entirely voluntary, and no incentives were provided.

### 3.2 Measures

#### 3.2.1 Loneliness

UCLA Loneliness Scale (UCLA-LS, Version 2) was used to measure students’ loneliness level (Russell, 1996). The scale consists of twenty questions that measure how often the participants feel disconnected from others. Participants were asked to rate on a 4-point Likert scale, ranging from *1 (never) to 4 (always)*, on the frequency of feeling lonely. Sample questions included “How often do you feel part of a group or friends?” and “How often do you feel that you are no longer close to anyone?” Reverse scoring was done for the positive statements. The sum of scores was calculated from the twenty questions. A higher score indicates a higher degree of loneliness. Individuals who score between 20 and 34 are considered to have a low level of loneliness, while those who score between 35 and 49 are considered to have a moderate level. Scores between 50 and 64 are classified as moderately high, and scores between 65 and 80 are considered high levels of loneliness. The Cronbach’s alpha values reported in the original study ranged from 0.89 to 0.94 (Russell, 1996), whereas the alpha coefficient obtained in the current sample was 0.70, indicating acceptable internal consistency.

#### 3.2.2 Perceived online social support

The Online Social Support Scale (OSSS) was developed by Nick et al. (2018) and was adopted in this study for measuring perceived online social support from students. The scale consists of forty statements that measure the level of online social support from the participants. Participants were asked to rate on a 5-point Likert scale, ranging from *0 (never) to 4 (a lot)*, regarding the frequency of the statements that happened to them while they interacted with others online over the last 2 months. Sample statements included “People show that they care about me online” and “Online, people make me feel like I belong.” The sum of scores calculated from the forty questions indicates the level of perceived online social support. A higher score indicates greater perceived online social support. In the original validation study of the OSSS, the four 10-item subscales demonstrated excellent internal consistency, with Cronbach’s alpha values of 0.95 for Esteem/Emotional Support, 0.94 for Social Companionship, 0.95 for Informational Support, and 0.95 for Instrumental Support (Nick et al., 2018). In the present study, the overall 40-item OSSS yielded a Cronbach’s alpha of 0.93, indicating high internal reliability for the total scale.

#### 3.2.3 Internet addiction

Internet Addiction Test (IAT) was used to measure the level of internet addiction in the participants (Young, 1998). The scale consists of twenty questions on problematic behaviors associated with excessive Internet use. Participants were asked to rate on a 6-point Likert scale, ranging from *0 (not applicable) to 5 (always)*. Sample questions included “How often do you find that you stay online longer than you intended?” and “How often do you lose sleep due to being online?” The sum of scores calculated from the twenty questions indicates the degree of internet addiction. A higher score indicates a higher level of internet addiction. Individuals who score between 0 and 30 are considered to have a normal level of internet usage, while those who score between 31 and 49 are considered to have a presence of mild internet addiction. Scores between 50 and 79 are classified as a moderate level of internet addiction, and scores between 80 and 100 are considered to be severe, dependent upon the internet. The IAT was recently validated among Chinese undergraduate students by Wei et al. (2025), who reported a Cronbach’s alpha of 0.93 for the overall scale, indicating excellent internal consistency. In the present study, the IAT demonstrated similarly strong reliability, with a Cronbach’s alpha of 0.90.

### 3.3 Data analyses

The 214 samples were tested for influential multivariate outliers using Mahalanobis’ Distances method (Mahalanobis, 1930). One outlier was identified and hence was not included in the data analysis, yielding a final sample of 213 for descriptive and influential statistical analysis. Pearson’s correlation was performed to test the association among the three outcome measures and age. Sex differences in all outcome measures were tested using an independent *t*-test. The Bonferroni correction method was adopted to control the effect of multiple comparisons.

To examine the conditional indirect and direct effects of loneliness on internet addiction through perceived online social support, a moderated mediation analysis was conducted using Model 59 of the PROCESS macro v4.2 for SPSS (Hayes, 2013). In this model, loneliness was specified as the independent variable, perceived online social support as the mediator, and internet addiction as the dependent variable. Sex was included as a moderator of both the path from loneliness to online social support and the path from online social support to internet addiction, while age was entered as a covariate. The analysis employed a nonparametric bootstrapping procedure with 5,000 resamples to generate bias-corrected 95% confidence intervals for inference. A significant effect was inferred when the confidence interval did not include zero (Preacher and Hayes, 2008). The model estimated conditional direct and indirect effects across levels of the moderator, as well as the index of moderated mediation. Descriptive and influential statistics were analyzed using SPSS v29. *p*-values less than 0.05 are considered statistically significant unless otherwise stated.

## 4 Results

The descriptive statistics and sex differences in age and the major outcomes are listed in Table 1. The majority of our participants had a moderate to moderately high level of loneliness and a mild to moderate level of addiction to internet usage. No significant differences in age, *t* (211) = −0.35, *p* = 0.724, loneliness, *t* (211) = −0.40, *p* = 0.691, perceived online social support, *t* (211) = −0.13, *p* = 0.895 and internet addiction levels, *t* (211) = −0.12, *p* = 0.904 were observed between male and female participants.

**Table 1:** Descriptives and sex differences in age and major outcomes.

Outcome variables	Total samples		Male		Female	
	Mean	SD	Mean	SD	Mean	SD
Age	21.63	2.35	21.57	2.55	21.69	2.14
UCLA-LS	48.84	8.83	48.59	8.43	49.07	9.24
IAT	46.95	13.74	46.84	12.93	47.06	14.54
OSSS	85.69	23.71	85.48	22.97	85.91	24.52

Total sample size = 213 (Male = 105; Female = 108). UCLA-LS, UCLA-Loneliness Scale; ITA, Internet Addiction Test; OSSS, Online Social Support Scale.

### 4.1 Pearson’s correlation analysis

For all participants, weak positive associations were observed between loneliness and internet addiction levels (*r* = 0.21, *p* = 0.002), and between loneliness and perceived online social support (*r* = 0.18, *p* = 0.007) (Table 2). A moderate positive association was observed between internet addiction level and perceived online social support (*r* = 0.31, *p* < 0.001). No significant associations were observed between age and the major outcome variables. A similar pattern was observed in the male participants (Table 2), but not in the female participants, except for the moderate positive association between internet addiction level and perceived online social support (*r* = 0.291, *p = 0*.002; Table 2).

**Table 2:** Pearson’s correlation among major outcomes and age.

	UCLA-LS		IAT		OSSS	
	*r*	*p*	*r*	*p*	*r*	*p*
A. Total samples						
Age	0.03	0.634	−0.02	0.767	0.04	0.556
UCLA-LS	–	–	0.21	0.002*	0.18	0.007*
IAT	–	–	–	–	0.31	< 0.001*
OSSS	–	–	–	–	–	–
B. Male						
Age	−0.07	0.502	0.04	0.663	0.12	0.213
UCLA-LS	–	–	0.28	0.004*	0.27	0.005*
IAT	–	–	–	–	0.33	< 0.001*
OSSS	–	–	–	–	–	–
C. Female						
Age	0.14	0.158	−0.09	0.372	−0.05	0.619
UCLA-LS	–	–	0.15	0.125	0.11	0.244
IAT	–	–	–	–	0.29	0.002*
OSSS	–	–	–	–	–	–

Total sample size = 213 (Male = 105; Female = 108). UCLA-LS, UCLA-Loneliness Scale; ITA, Internet Addiction Test; OSSS, Online Social Support Scale. *Bonferroni corrected *p*-value = 0.05/6 = 0.008.

### 4.2 Moderated mediation analysis

**Figure 1:**
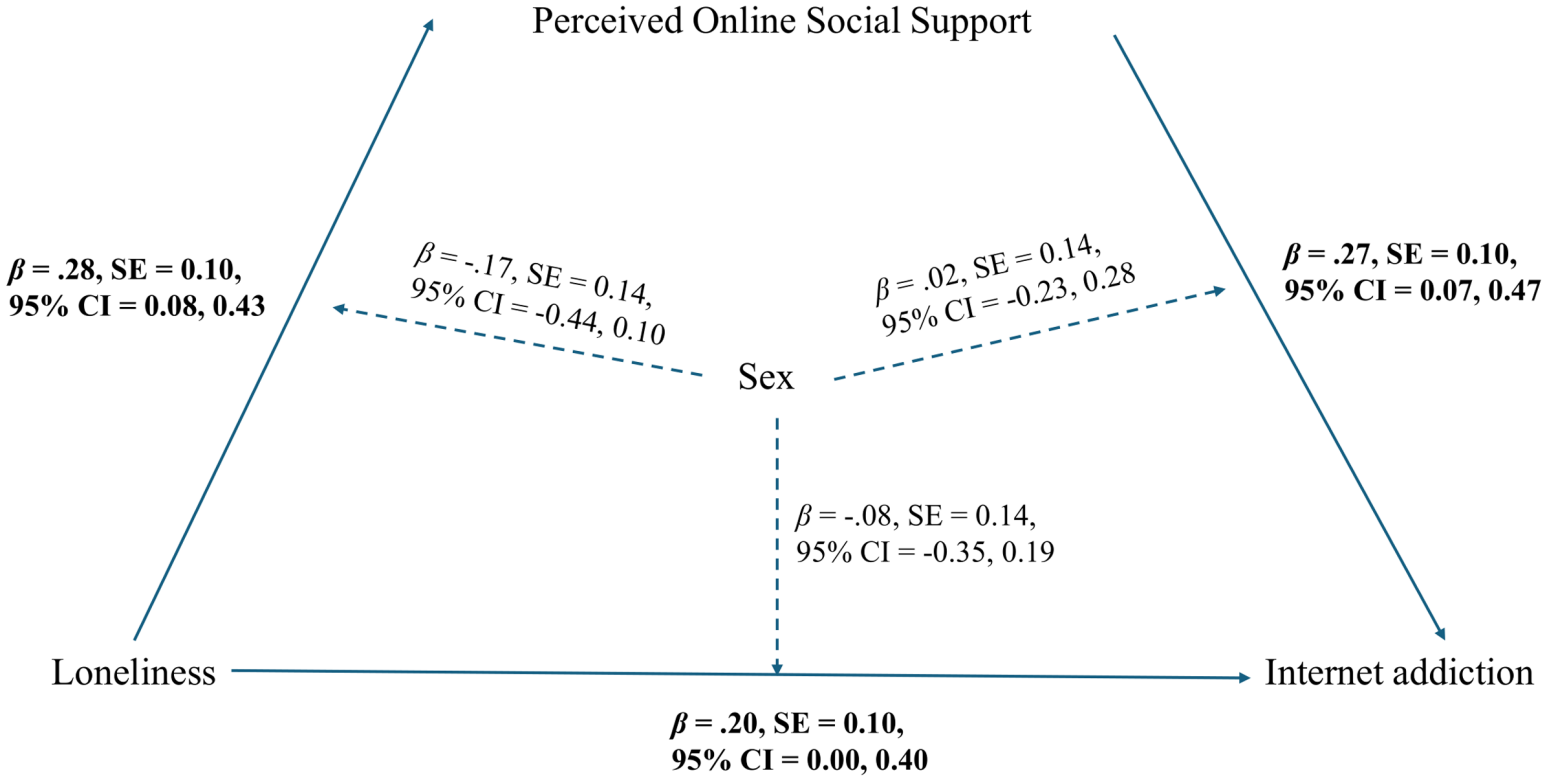
Moderated mediation of loneliness on internet addiction via online social support, moderated by sex. Conditional process model illustrating the direct and indirect effects of loneliness on internet addiction via perceived online social support, moderated by sex. Standardized path coefficients are shown. Bootstrapped standard errors and 95% confidence intervals are presented. Statistically significant paths are shown in bold.

A moderated mediation analysis was conducted with loneliness as the independent variable, perceived online social support as the mediator, internet addiction as the dependent variable, and sex as a moderator of the direct as well as both mediation paths. Age was included as a covariate. In the first stage, loneliness was significantly associated with perceived online social support (*p* = 0.007), although the overall model was marginally significant, *F* (4, 208) = 2.30, *p* = 0.060, and the interaction between loneliness and sex was not statistically significant. In the second stage, the overall model regressing internet addiction was statistically significant, *F* (6, 206) = 4.70, *p* < 0.001. Within this model, both loneliness (*p* = 0.048) and perceived online social support (*p* = 0.009) were significant regressors of internet addiction, while neither interaction term (sex × loneliness, sex × online social support) reached significance (*ps* > 0.05). The moderated mediation model with standardized path coefficients and bootstrapped confidence intervals is presented in Figure 1.

Conditional effects analysis revealed that the direct effect of loneliness on internet addiction was significant for males [standardized *β* = 0.20, bootstrapped SE = 0.10, *p* = 0.048, bootstrapped 95% CI (0.00, 0.40)], but not for females [standardized *β* = 0.12, bootstrapped SE = 0.09, *p* = 0.170, bootstrapped 95% CI (−0.05, 0.30)]. Similarly, the indirect effect of loneliness on internet addiction via perceived online social support was significant for males [standardized *β* = 0.07, bootstrapped SE = 0.04, bootstrapped 95% CI (0.01, 0.17)], but not for females [standardized *β* = 0.03, bootstrapped SE = 0.03, bootstrapped 95% CI (−0.03, 0.11)]. The index of moderated mediation was −0.04, with a bootstrapped 95% confidence interval of [−0.15, 0.06], indicating that the difference in indirect effects between males and females was not statistically significant.

## 5 Discussion

Our findings confirmed the previous observation that loneliness is positively linked to internet addiction, and further suggested that this positive relationship was partially mediated through perceived online social support in male participants. Although the interaction terms between sex and both loneliness and perceived online social support were not statistically significant, the conditional effects analysis revealed sex-specific direct and indirect effects. For males, the direct effect of loneliness on internet addiction was significant, indicating that higher levels of loneliness were associated with increased internet addiction independent of perceived online social support. The indirect effect was also significant for males, suggesting that loneliness contributed to greater internet addiction in part through increased perceived online social support. In contrast, neither the direct nor indirect effects were statistically significant for females, implying that the pathway from loneliness to internet addiction may differ across sexes.

The positive relationship between loneliness and internet addiction in our young male sample largely corroborates previous literature (Ge et al., 2025). In a recent meta-analysis of thirty-two empirical studies that involved a total of 35,623 adolescents and adults, a moderately positive association between loneliness and internet addiction was confirmed. The positive association was moderated by objective demographic variables like age, sex, and region of study (Wang and Zeng, 2024), suggesting the association between loneliness and internet addiction can vary among different populations. For instance, the correlation strength between loneliness and internet addiction was higher in university students than in adolescents, in male than female participants, and in East and West Asian countries than in European countries such as Greece (Wang and Zeng, 2024). A more recent study also reported an association between social and emotional loneliness and internet addiction in Turkish university students (Ataman-Bor et al., 2025). Results from another study further clarified the directions of the associations between loneliness and internet addiction in 220 Chinese post-secondary students using a longitudinal cross-lagged analysis approach (Zhang et al., 2018). Specifically, internet addiction problems at baseline significantly and positively predicted loneliness levels at the 6-month timepoint; loneliness levels at the 6-month timepoint also significantly and positively predicted internet addiction problems at the 1-year timepoint. More importantly, loneliness had a greater and more extensive effect on internet addiction than the reverse.

Compared to our male participants, the association between loneliness and internet addiction was weaker and not statistically significant among female participants. However, these sex differences also did not reach statistical significance, which may be attributed to the limited sample size in this study. In fact, associations between loneliness and internet addiction in female subjects were inconsistent in different populations around the world. Turan et al. (2020) did not observe any association between loneliness and internet addiction in 160 nursing students in Turkey, who are primarily female students (93.1%). In contrast, Rachubińska et al. (2021) found positive associations between loneliness and internet / Facebook addiction in Poland women. Another study that was conducted on only female patients with acne vulgaris also found a positive association between loneliness and internet addiction; the levels of loneliness, internet addiction, and depression in the acne vulgaris group were all higher than those in the control group (Öztekin and Öztekin, 2020). It is possible that the distress and depressive mood associated with acne vulgaris may have acted as a third variable that heightened both the feeling of loneliness and internet addiction in their female sample. The absence of loneliness-internet addiction linkage in our female students may be related to the different purposes of internet use between males and females. Previous studies reported that online gaming was the main internet use in males (i.e., leisure use), while females mainly used the internet for social communication (i.e., social use; Lavoie et al., 2023; Wang et al., 2023). For instance, online gaming is a highly appealing stimulus that can only be accessed through the internet. As a result, males tend to spend time on online games for self-actualization and personal needs (Dufour et al., 2016), rendering them more vulnerable to problematic internet use. Conversely, females tend to spend time online for social and interpersonal needs, which can also be fulfilled through interpersonal activities in the real world, thus alleviating their loneliness without solely depending on the internet (Wang et al., 2023). It is noteworthy to mention that due to the limited sample size in this study, the null findings could also be due to insufficient power for detecting the small effect. Future studies with larger samples are required to confirm our findings.

While sex differences in internet addiction problems are well-documented in the literature, what is less clearly known is the psychosocial mechanism underlying the loneliness-internet addiction relationship, especially in young men. Our findings contributed to the existing literature by suggesting that the perception of online social support is involved in the positive association between loneliness and internet addiction in young men in Hong Kong. From our results, online social supports perceived by lonely young men were associated with higher levels of internet addiction. This contrasts with the traditional view that social support helps to reduce the feeling of loneliness and improve mental health conditions. For instance, a recent meta-analysis demonstrated a negative association between loneliness levels and perceived social support in real life (Zhang and Dong, 2022), suggesting that people who perceive fewer actual social support feel lonelier, whereas people who have more actual social support are less lonely. Findings from a more recent study echoed these points by suggesting that family support had a negative association with loneliness, internet addiction, and depression in children and adolescents (Zhou et al., 2025).

In contrast, another recent meta-analytic and systematic review study provided some initial evidence on the beneficial effect of online social support on self-esteem in adolescents, but not on depression (Zhou and Cheng, 2022). They highlighted an important distinction between the beneficial effects of real-life and online social support. Although the potential benefits of online social support may seem promising in this digital era, social support from acquaintances on the internet was considered not to be as beneficial as from real-life acquaintances (Zhou and Cheng, 2022). In fact, when lonely individuals, who constantly crave for connectedness and social support (Saporta et al., 2021), go online, their internet use, including online gaming that involves peer support groups and online social communication channels, could potentially enhance the perception of social support and ease the feeling of loneliness (Luo et al., 2022). However, such a relieving effect could be temporary and does not have a strong effect on one’s mental health condition (e.g., non-significant association between online social support and depression in adolescents; Zhou and Cheng, 2022). More specifically, in-game online social support from online gaming networks was found to be unrelated to symptoms of depression and anxiety in young adults; alternatively, only real-life social support was found to be negatively correlated with these symptoms (Tham et al., 2020). The unresolved and persisting desire to receive more social support could result in more support-seeking behaviors on the internet and eventually more internet addiction. According to the literature, increased use of the internet and internet addiction were associated with social withdrawal from the real world (Tateno et al., 2019), which in turn could elevate feelings of loneliness (Bowker et al., 2019). Previous studies that contrasted the effect of offline and online social support showed that internet addiction negatively correlated with offline social support but positively with online social support (Lin et al., 2018; Liu et al., 2021; Wang and Wang, 2013; Yeh et al., 2008). People who were diagnosed as internet addicts had a higher tendency to escape from reality and turn to others on the internet in times of need and showed an unusually close feeling for strangers (Whang et al., 2003). However, it is difficult to maintain virtual social relationships as they can be easily broken (Lin et al., 2018), and online social support was not found to improve young adults’ mental well-being (Longest and Kang, 2022). Following this line of thought, it is plausible that online social support may not necessarily be beneficial to one’s mental health; instead, it may have some contribution to a deeper reliance on the internet, especially in young men. Given that there are different ways to operationalize online social support (e.g., different sources such as family and friends versus online acquaintances, different functions such as emotional versus informational, and so on), our findings need further replication and verification to understand more about the benefits and/or problems associated with online social support received or perceived from different sources and/or with different functions. Future studies should also consider the effects of different types of internet use, e.g., online gaming and social communication platforms, to further elucidate the sex effect on this issue.

### 5.1 Limitations

There are several limitations in this study. First, the cross-sectional design of this study limited our ability to examine the directionality of the relationships among loneliness, perceived online social support, and internet addiction. Although the mediation findings were statistically robust, causal inferences cannot be drawn. Additionally, the use of self-report measures may have introduced bias, including social desirability bias, which could lead participants to underreport behaviors perceived as problematic or overreport socially acceptable attitudes. These limitations highlight the need for future research employing longitudinal or experimental designs, as well as incorporating multi-method and longitudinal approaches to reduce bias and strengthen causal interpretations. Second, some confounding variables, such as types of internet use (i.e., gaming vs. communication), mental health conditions (e.g., depression), and offline social support, were not captured in this study, which limited the interpretation of our findings. Future studies should include these variables to more comprehensively understand the role of social support, be it received online and offline, on the relationship between loneliness and internet addiction. Third, this study relied on a convenience sample of university students in Hong Kong, which may introduce sampling bias and limit the generalizability of the findings to other populations. University students represent a specific demographic, typically younger, more digitally engaged, and embedded within a particular cultural and educational context, which may not reflect the experiences of individuals from other age groups or regions. Consequently, the observed relationships between loneliness, perceived online and offline social support, and internet addiction may differ across populations with varying social environments and internet usage patterns. Replication studies in other countries and among more diverse samples are needed to assess the broader applicability of these findings. Fourth, the relatively small sample size in this study may have limited the statistical power to detect complex effects within the moderated mediation model. As such, the interpretation of our findings should be approached with caution. Future research employing larger and more diverse samples is recommended to validate and extend these results. Last, to the best of our knowledge, this is the first study to apply the full 40-item OSSS in a Chinese-speaking sample. While we employed the original English version due to the high English literacy of our university student participants in Hong Kong, we acknowledge that cultural differences may influence how online social support is interpreted and expressed. Future research should consider localized adaptations and qualitative validation to enhance the scale’s cross-cultural applicability and conceptual relevance.

## 6 Conclusion

Our findings suggest that online social support may play a complex role in the relationship between loneliness and internet addiction, particularly among young men. This raises important considerations for mental health professionals and educators when designing interventions, highlighting the need to differentiate between online and offline support systems and to address the potential risks of increased reliance on digital interactions as a coping mechanism for loneliness. This deserves more in-depth investigations regarding different online social support received or perceived through different sources and different online platforms and environments. Future studies should employ longitudinal or experimental designs and multi-method approaches to clarify causal relationships among loneliness, online social support, and internet addiction, while also accounting for confounding variables such as internet use type, mental health status, and offline support. Additionally, replication studies across diverse populations and cultural contexts are essential to enhance generalizability and cross-cultural relevance.

## Data Availability

The original contributions presented in the study are included in the article/supplementary material, further inquiries can be directed to the corresponding authors.
